# A Latent profile and network intervention analysis of psychosomatic symptoms in lung cancer immunotherapy patients

**DOI:** 10.1371/journal.pone.0357460

**Published:** 2026-09-09

**Authors:** Danwen Zheng, Hang Gao, Yanfei Zou, Qiuyue Shao, Xinyan Yu, Lanying Qiu, Xiaoli Sun

**Affiliations:** 1 Phase I Clinical Ward, Zhejiang Cancer Hospital, Hangzhou, Zhejiang, China; 2 Department of Thoracic Medicine, Zhejiang Cancer Hospital, Hangzhou, Zhejiang, China; 3 Postgraduate Training Base Alliance of Wenzhou Medical University (Zhejiang Cancer Hospital), Hangzhou, Zhejiang, China; 4 Department of Thoracic Radiotherapyl, Zhejiang Cancer Hospital, Hangzhou, Zhejiang, China; Hokkaido University: Hokkaido Daigaku, JAPAN

## Abstract

**Background:**

Immune checkpoint inhibitors improve non-small cell lung cancer prognosis but induce complex, heterogeneous psychosomatic symptoms. Traditional symptom-focused approaches fail to capture underlying patient heterogeneity and interaction mechanisms.

**Methods:**

In this cross-sectional study of 971 lung cancer patients undergoing immunotherapy, symptom phenotypes were identified using Latent Profile Analysis. Influencing factors were analyzed via multivariate logistic regression. Multivariate logistic regression was used to analyze influencing factors, and symptom networks and optimal intervention targets were investigated using network analysis and computer-simulated interventions.

**Results:**

The study identified three phenotypes: “High Symptom Burden with Comorbid Distress”(C1, 25.1%), “Emotional Distress Dominant”(C2, 29.2%), and “Mild Adaptive”(C3, 45.6%). Logistic regression (FDR‑adjusted q < 0.05) revealed that smoking history (OR=28.1, 95% CI: 5.2–151.8), clinical stage IV, and poor ECOG performance status (OR range: 5.9–8.4) were strong risk factors for the high‑burden phenotype (C1), while combination therapy (OR=0.22, 95% CI: 0.08–0.56), living with family (OR=0.15, 95% CI: 0.05–0.44), and absence of tumor metastasis were protective. Female sex (OR=3.3) was a risk factor for C2. Network analysis revealed core symptoms and bridging symptoms specific to each phenotype: Fatigue was the core symptom for C1, with interference in general activities serving as the bridging symptom; C2’s core symptom was sleep disturbance, with loss of interest serving as the bridging symptom; in C3, diminished enjoyment of life functioned as both core and bridging symptom. Based on cross‑sectional data, computer simulations further suggested potential intervention targets for each phenotype: fatigue for C1, enjoyment of life for C3, and interpersonal networks for C2. Network density was significantly higher in C1 and C2 than in C3 (both *p* < 0.001).

**Conclusions:**

This study highlights the complex interplay between somatic symptoms and anxiety‑depression in lung cancer immunotherapy patients, offering hypothesis‑generating evidence for precise intervention pathways tailored to distinct symptom phenotypes. It provides preliminary insights toward advancing symptom management toward stratified, personalized intervention models.

## 1 Introduction

Lung cancer is the leading cause of cancer-related deaths worldwide, claiming approximately 1.8 million lives annually. Non-small cell lung cancer (NSCLC) accounts for over 85% of all cases, constituting the primary disease burden [1–3]. Taking China as an example, its annual incidence represents about 36% of the global total, posing persistent challenges for disease prevention and control [4].Despite advances in treatment, the 5-year overall survival rate for patients with advanced NSCLC remains below 20%, highlighting the urgent need to improve prognosis [5]. The application of immune checkpoint inhibitors (ICIs), exemplified by programmed death receptor-1 (PD-1)/programmed death-ligand-1 (PD-L1) inhibitors, has significantly improved survival outcomes for advanced NSCLC patients. For instance, pembrolizumab monotherapy has elevated the five-year survival rate to 31.9% in PD-L1-positive patients [6,7]. However, unique pathophysiological alterations induced by ICI therapy frequently lead to cancer-related fatigue, immune-related adverse events (irAEs), and a complex array of psychosomatic symptoms including anxiety and depression [8].These symptoms do not exist in isolation but interact through neuro-endocrine-immune pathways, forming a dynamic symptom network that synergistically compromises patients’ treatment tolerance, adherence, and health-related quality of life.

Research indicates that lung cancer patients receiving immune checkpoint inhibitor (ICI) therapy frequently experience multiple coexisting physical and psychological symptoms. For instance, approximately 75% of patients report moderate to severe fatigue, 33.0%−77.2% exhibit anxiety or depressive symptoms, and 49% experience sleep disturbances [9–11]. These symptoms do not exist in isolation; their synergistic effects significantly impair patients’ physical functioning, mental health, and clinical outcomes. However, previous studies have largely been limited to analyzing single symptoms or evaluating predefined symptom clusters. While such approaches can reflect overall symptom burden through total scale scores, they struggle to reveal individual differences in symptom presentation across patient populations or the complex dynamic relationships between symptoms [12]. This methodological limitation directly hinders the clinical translation and application of individualized symptom management strategies. Identifying symptom heterogeneity is a crucial foundation for achieving precision medicine and personalized care. Symptom patterns among patients are influenced by multiple factors, including tumor stage, immune response status, and psychological resilience, and are closely associated with varying clinical outcomes [12–14]. Therefore, it is imperative to transcend traditional research frameworks and delve deeper into the individualized expression patterns of symptoms and their underlying mechanisms. This will enable the provision of more targeted, evidence-based nursing interventions for lung cancer patients receiving ICIs.

Latent Profile Analysis (LPA) is a person-centered approach that has become a key tool for identifying heterogeneous patient subgroups [15]. By categorizing individuals into optimal profiles, it is widely applied in health psychology and oncology research for symptom phenotype identification and prognostic stratification [16–18]. Additionally, Symptom Network Analysis (NA) is an innovative paradigm that conceptualizes psychosomatic symptoms as a dynamic, interactive network [19].Unlike traditional linear approaches, NA characterizes the associative structure among symptoms to identify core symptoms (hubs driving the network) and bridge symptoms (connecting different symptom dimensions) [20–22]. By identifying these key nodes, NA provides precise guidance for selecting priority targets in symptom management.

In recent years, NA has gradually been applied to the field of oncology nursing, offering a novel perspective for deciphering the underlying mechanisms of complex symptoms in cancer patients. However, existing research has primarily focused on patients receiving conventional therapies (chemotherapy, radiotherapy), with limited exploration of symptom network characteristics in immunotherapy-treated lung cancer patients. Conventional chemotherapy causes systemic cytotoxic damage to rapidly proliferating normal tissues [23], leading to predictable side effects such as bone marrow suppression, gastrointestinal reactions, and hair loss; radiotherapy, on the other hand, causes localized ionizing radiation damage to normal tissues within the treatment field [24], resulting in region-specific symptoms such as radiation dermatitis, pneumonitis, and enteritis. Although their mechanisms differ, both primarily involve unidirectional, direct tissue damage, and their symptoms are relatively predictable. In contrast, the pathophysiological mechanisms underlying symptoms in patients treated with immune checkpoint inhibitors (ICIs) exhibit two fundamental differences. First is the systemic immune-mediated mechanism. ICIs exert their therapeutic effects by lifting immune suppression and activating a systemic antitumor immune response; this process may disrupt the body’s immune homeostasis, leading to abnormal T-cell activation and inducing immune-related adverse events (irAEs) involving multiple organs [25]. Compared to the localized or systemic cytotoxic effects of chemoradiotherapy, ICI-related symptoms are typically immune-mediated, unpredictable in onset, and highly heterogeneous across individuals. Second, there is a unique bidirectional mind-body pathophysiological mechanism. Existing studies confirm that negative emotions and psychological distress can suppress the body’s antitumor immune function through psychoneuroimmunological pathways; for example, a study by Zeng et al. [26] clearly demonstrated that baseline emotional distress significantly weakens the efficacy of ICI therapy in patients with non-small cell lung cancer. Conversely, ICI-induced systemic immune inflammatory responses and irAEs exacerbate patients’ physical symptoms, thereby inducing or worsening psychological distress such as anxiety and depression. This complex bidirectional vicious cycle, “psychological distress - immune dysfunction - symptom exacerbation - reduced treatment response”, is the core reason why symptom heterogeneity is significantly higher in ICI patients compared to those receiving radiotherapy or chemotherapy.

Given these mechanistic differences, patients receiving ICI therapy exhibit heterogeneous phenotypes of psychosomatic symptoms, implying that clinical intervention strategies must be tailored to the specific phenotype. Existing studies have identified multiple independent symptom clusters in ICI-treated patients, including emotional disturbances, lung cancer-specific somatic symptoms, and skin toxicity [101]. Therefore, rather than applying symptom management protocols universally applicable to patients undergoing chemoradiotherapy, specific intervention targets for different phenotypes should be identified. For instance, a phenotype dominated by somatic toxicity may require targeted immunomodulation or irAE management, whereas a psychological distress-dominated phenotype would benefit primarily from psycho-neuro-immunological interventions (e.g., cognitive behavioral therapy). Based on this, this study identifies heterogeneous psychophysical symptom phenotypes in ICI-treated patients through latent profile analysis, further distinguishes the clinical and psychosocial predictors of each phenotype, constructs specific symptom networks, and explores potential intervention targets for each phenotype using computer simulation methods, aiming to provide a reference for phenotype-based, differentiated, and precise symptom intervention in ICI patients.

## 2 Materials and methods

### 2.1 Design and participants

February 2024–October 2025: Lung cancer patients admitted to Phase I wards, thoracic radiotherapy wards, and thoracic medicine wards at Zhejiang Cancer Hospital were selected as study subjects using convenience sampling. Inclusion Criteria: (1) Age ≥ 18 years, conscious and able to communicate effectively; (2) Pathologically confirmed primary bronchogenic lung cancer, with TNM staging classified as Stage III or IV; (3) Previous immunotherapy treatment with immune checkpoint inhibitors (ICIs) for ≥1 month; (4) Voluntary signing of informed consent to participate in this study. Exclusion criteria: (1) Presence of severe psychiatric disorders (e.g., schizophrenia, acute phase of major depressive disorder) rendering the patient unable to complete questionnaire surveys; (2) Concurrent severe underlying conditions (e.g., severe heart failure, end-stage renal disease) or other primary malignancies; (3) Cognitive impairment or language communication barriers. Data collection and quality control: Data were collected using an electronic questionnaire. The introductory section of the questionnaire detailed the study purpose, significance, and completion instructions. After each logical branching, all items were set as mandatory, and each IP address was allowed to submit the questionnaire only once. Consequently, there were no missing data at the item level, obviating the need for imputation in the subsequent latent profile analysis (LPA) and network analyses.

### 2.2 Sample size

The required sample size was determined to satisfy both multivariate and network analyses. For multivariate analysis, based on the recommendation of 5–10 cases per independent variable (n = 33), a minimum of 165–330 patients was needed [27]. For symptom network analysis, the minimum requirement of P(P-1)/2 (where P = 33 symptom nodes) necessitated at least 528 patients to ensure statistical power [28]. The final enrolled sample of 971 patients effectively met the requirements of both analytical approaches.

### 2.3 Ethics

This study strictly adheres to the ethical principles of the Declaration of Helsinki and has been approved by the Ethics Committee of Zhejiang Cancer Hospital (IRB-2024–149). All participants voluntarily signed informed consent forms after being fully informed of the study’s purpose, procedures, and risks, and may withdraw at any time. Throughout the study, participant privacy and data security are rigorously protected, with all information used solely for research purposes.

### 2.4 Instrument

#### 2.4.1 General information questionnaire.

A self-designed questionnaire collected sociodemographic characteristics (age, gender, education, income, living arrangements, and medical payment) and clinical data (tumor stage, immune-related adverse events (irAEs), treatment modality, metastasis, ECOG performance status, smoking and drinking history, and exercise habits). Age was categorized into three groups based on established geriatric oncology standards and previous literature: ≤ 59 years (non-elderly), 60–74 years (younger older adults), and ≥75 years (older elderly). This classification combines the World Health Organization’s definition of older adults (≥60 years) [29] with the commonly used cutoff age of 75 years in expert consensus statements on the treatment of advanced lung cancer in older adults [30].

#### 2.4.2 MD anderson symptom inventory (MDASI).

The MDASI scale [31,32]. was used to assess patients’ burden of cancer-related symptoms. The scale consists of two sections: (1) symptom severity (13 items, assessing core physical and psychological symptoms such as pain, fatigue, and sleep disturbances); and (2) functional impairment (6 items, assessing the impact of symptoms on daily activities and mood). All items are scored on a 0–10 scale, where 0 indicates “no symptoms/no interference” and 10 indicates “most severe symptoms/complete interference.” Higher scores indicate a heavier symptom burden. The scale has been extensively validated in cancer populations, demonstrating excellent internal consistency (Cronbach’s alpha 0.89–0.94) and test-retest reliability (ICC ≥ 0.85) [31,33].

#### 2.4.3 Hospital anxiety and depression scale (HADS).

This study used the HADS scale [34] to assess patients’ psychological distress. Designed to minimize the confounding effects of physical symptoms in clinical populations, the scale consists of 14 items divided into two independent 7-item subscales: anxiety (HADS-A) and depression (HADS-D). Each item is scored on a scale of 0–3, and the total score for each subscale ranges from 0 to 21. Subscale scores ≥8 indicate the presence of clinically significant anxiety or depressive symptoms. The HADS has demonstrated good reliability in patients with various types of cancer, with Cronbach’s α coefficients of 0.86 for the anxiety subscale, 0.85 for the depression subscale, and 0.91 for the total scale [35].

### 2.5 Statistical analyses

#### 2.5.1 Latent profile analysis.

The tidyLPA package in R 4.3.0 was employed to analyze the latent profile structure of psychosomatic symptoms. Beginning with a single-profile model, we incrementally increased the number of profiles. Model fit was evaluated using the following criteria to determine the optimal model: (1) Information Criteria: the Akaike Information Criterion (AIC), Bayesian Information Criterion (BIC), and adjusted BIC (aBIC), with lower values indicating better fit; (2) Classification Metric: entropy (ranging from 0 to 1), where values closer to 1 denote higher classification accuracy (entropy ≥0.80 corresponds to >90% accuracy); (3) Likelihood Ratio Test: the Bootstrap Likelihood Ratio Test (BLRT) was used to compare fit between models; a significant result (*P* < 0.05) indicated that model k provided a better fit than model k-1 [36]. While these statistical indices served as key references, the final model selection also incorporated the substantive interpretability of each profile.

#### 2.5.2 Analysis of general characteristics.

All statistical analyses were performed using R software (V.4.3.0). Categorical variables were described using frequencies and percentages (or proportions). To assess the potential impact of common-method bias, we performed Harman’s single-factor test using unrotated principal component analysis on all 33 items of the MDASI and HADS. To identify the predictors most strongly associated with latent categories of psychosomatic symptoms in lung cancer patients receiving immunotherapy, this study performed multiple LASSO (Least Absolute Shrinkage and Selection Operator) regression analyses using the glmnet package in R. All candidate variables were included as predictors in the model, with the dependent variable being a trichotomous variable (i.e., the three symptom phenotypes identified by latent profile analysis). To ensure that each predictor was simultaneously included or excluded across all categories, the model applied grouped penalization to all outcome categories. The optimal regularization parameter λ was determined via 10-fold cross-validation, and variable selection was performed using the λ.min criterion, which minimizes the cross-validation classification error rate: variables with non-zero coefficients were retained for subsequent analysis. Multinomial logistic regression was performed with the symptom phenotype classifications obtained from LPA as the dependent variable (a three-class factor) and the variables selected by LASSO as independent variables. Multiple comparisons were corrected using the Benjamini-Hochberg false discovery rate (FDR) method. The significance level was set at α = 0.05.

#### 2.5.3 Correlation analysis.

To visualize the correlation patterns between symptom severity (MDASI), interference with daily life, and emotional distress (HADS anxiety and depression levels), a heatmap was generated using the ggcorrplot package in R 4.3.0 [37].

#### 2.5.4 Network estimation and visualization.

Symptom network analysis was performed using R 4.3.0. A symptom association network was constructed based on a Gaussian Graphical Model (GGM). Conditional associations between symptoms were quantified using a Spearman’s partial correlation matrix. Network sparsification was achieved via the graphical Least Absolute Shrinkage and Selection Operator (LASSO), and model parameters were optimized using the Extended Bayesian Information Criterion (EBIC) to identify statistically significant core associations. The network was visualized using the qgraph package [38], where nodes represent individual symptoms and edge thickness reflects the absolute value of the association strength. Network centrality indices-including strength, closeness, betweenness, and expected influence-were calculated. Among these, strength centrality, which demonstrates the highest stability in symptom network research [39], was designated as the primary indicator for identifying core symptoms. It is defined as the sum of the absolute edge weights connecting a node to all other directly connected nodes; a higher value indicates greater importance of the symptom within the network. Network stability and accuracy were assessed using the bootnet R package. Non‑parametric bootstrap (1,000 resamples) was used to calculate 95% confidence intervals for edge weights and node strength centrality. To evaluate the stability of centrality indices (strength, expected influence, betweenness, and closeness), case‑dropping subset bootstrap (1,000 iterations) was performed. Correlation stability (CS) coefficients were computed for each centrality index. Following established guidelines, CS > 0.25 was considered acceptable and CS > 0.5 indicated good stability. Finally, the NetworkComparisonTest package was used to compare subgroup networks, examining differences in network structure invariance, global strength, and specific edge weights. Differences with *P* < 0.05 were considered statistically significant.

#### 2.5.5 Computer-Simulated Network Intervention.

Model estimation and data simulation were performed using the R packages ‘bootnet’, ‘qgraph’, and ‘nodeIdentifyR’. From the estimated model parameters, we generated 15,000 synthetic participants and implemented two simulation conditions on the network: a strengthening (aggravating) intervention and a weakening (alleviating) intervention. According to the NodeIdentifyR algorithm [40], the intervention intensity was defined as two standard deviations of the threshold estimates. More specifically, after the network model was estimated, thresholds for all symptoms were extracted and their standard deviation was calculated. This value was then used to individually shift each symptom’s threshold parameter upward (aggravation) or downward (alleviation). Because the underlying model is non-linear, this standardized, data-driven approach, as opposed to a fixed absolute change, ensures that intervention effects can be compared meaningfully across symptoms with differing baseline thresholds. The algorithm recalculates network activity under the modified parameters, computes the shift in overall symptom activation relative to the baseline, and pinpoints nodes predicted to have the greatest impact on network dynamics [41]. These results are in-silico projections derived from the model and should be viewed as hypothesis-generating. Prospective longitudinal or experimental studies are needed to empirically validate these findings.

## 3 Results

### 3.1 LPA model fitting and final phenotype selection

This study employed 33 items from the MDASI and HADS for latent profile analysis. As shown in Table 1, while information criteria (AIC, BIC, SABIC) continuously decreased and likelihood ratio tests (BLRT, LMRT) remained statistically significant (*p* < 0.01) up to five classes, a notable “elbow” in the rate of BIC reduction appeared at the three-class model. Specifically, the decrease in BIC dropped sharply from ΔBIC = −4,541 (2-class vs. 3-class) to ΔBIC = −540 (3-class vs. 4-class), indicating diminishing returns for additional complexity. Furthermore, the three-class model demonstrated higher entropy (0.99) and more robust class proportions (smallest class = 25%), thereby avoiding the smaller, potentially less stable subgroups (each accounting for 13%) generated by the four-class and five-class models. The average posterior probabilities for the three-class solution were 0.994 (Class 1), 0.993 (Class 2), and 1.000 (Class 3), indicating excellent classification certainty. Crucially, the latent profile plots (S1 Fig) show that the three-class solution yields clearly distinguishable and clinically meaningful symptom patterns, high symptom burden, emotional distress, and mild adaptive, each with a clear clinical interpretation. In contrast, the additional categories in the four- and five-class solutions do not represent novel phenotypes. Balancing statistical parsimony with clinical interpretability, the three-class model was selected as the optimal solution.

**Table 1:** Model fitting metrics for the psychosomatic symptom profile model in lung cancer patients receiving immunotherapy.

Classes	LogLike	AIC	BIC	SABIC	Entropy	LMRT_p	BLR_p	Posterior Probability	Category Proportion
1	−66790.51	133713.02	134034.99	133825.37	1.00	–	–	–	–
2	−54285.30	108836.60	109485.42	109063.00	1.00	<0.01	<0.01	0.999/0.905	0.54/0.46
**3**	**−51784.37**	**103968.73**	**104944.40**	**104309.20**	**0.99**	<**0.01**	<**0.01**	**0.994/0.993/1**	**0.25/0.29/0.46**
4	−51283.72	103101.44	104403.96	103556.00	0.98	<0.01	<0.01	0.986/0.985/0.998/1	0.13/0.13/0.29/0.46
5	−50501.67	101671.35	103300.71	102239.90	0.97	<0.01	<0.01	0.984/0.989/0.999/0.980/0.983	0.13/0.13/0.28/0.30/0.16

**Fig 1:**
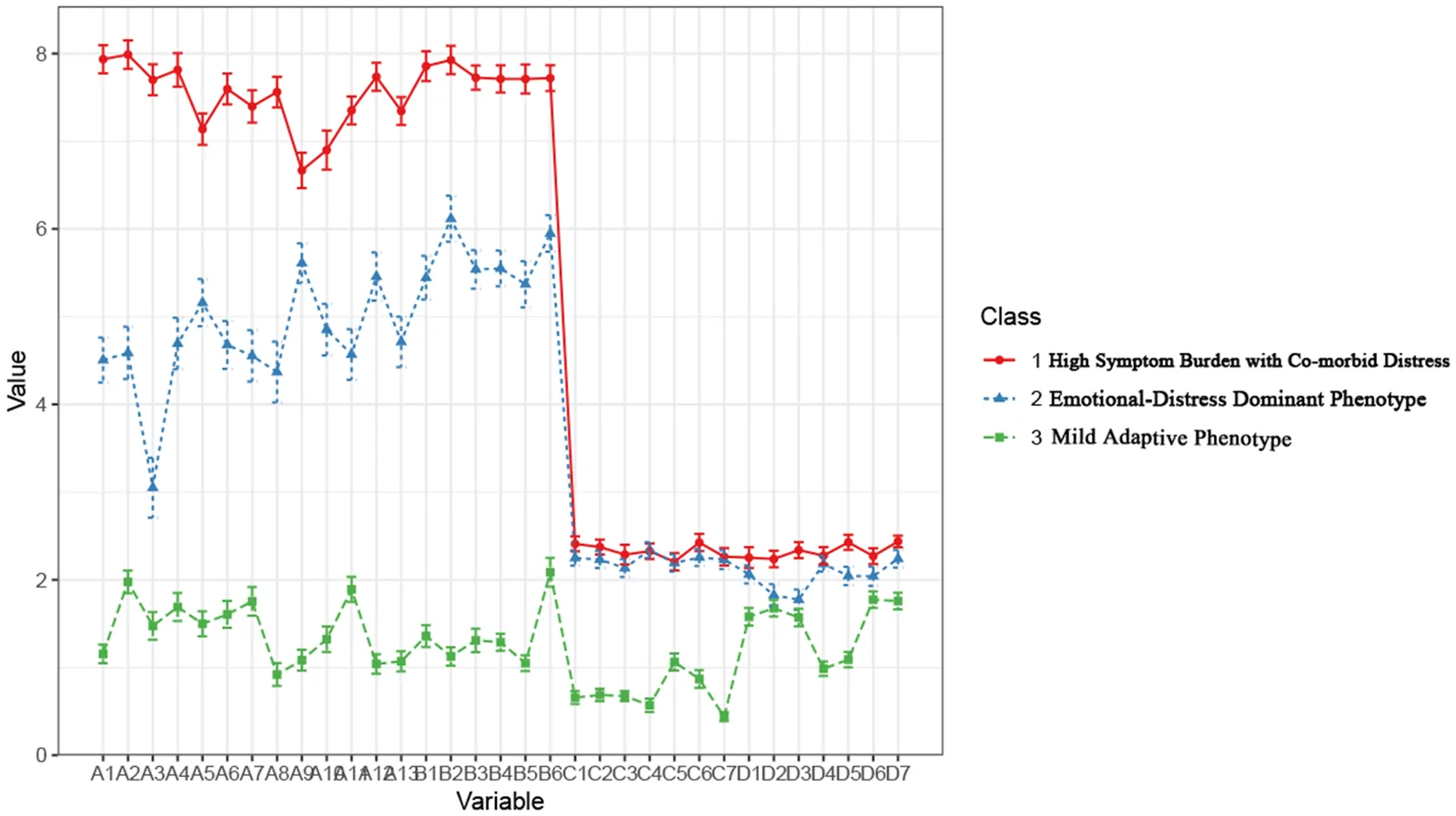
Characteristic distribution of three potential profiles of psychosomatic symptoms in lung cancer immunotherapy patients.

Based on the three-category model, the latent profiles were visualized (Fig 1) and characterized as follows:Category 1 (C1): High Symptom Burden with Co-morbid Distress (n = 244, 25.1%). This subgroup showed consistently high scores across all MDASI and HADS items, reflecting severe physical symptoms along with pronounced psychological distress.Category 2 (C2): Emotional-Distress Dominant Phenotype (n = 284, 29.2%). Characterized primarily by psychological distress, this group presented moderate physical symptom burden but notably elevated scores in emotional distress (B2) and loss of enjoyment in life (B6), accompanied by persistently high HADS total scores.Category 3 (C3): Mild Adaptive Phenotype (n = 443, 45.6%). This subgroup displayed generally low scores across both MDASI and HADS items, indicating mild symptom burden and favorable psychological adaptation.

### 3.2 Clinical characteristics across different phenotypes

#### 3.2.1 Baseline characteristics and LASSO regression.

**Fig 2:**
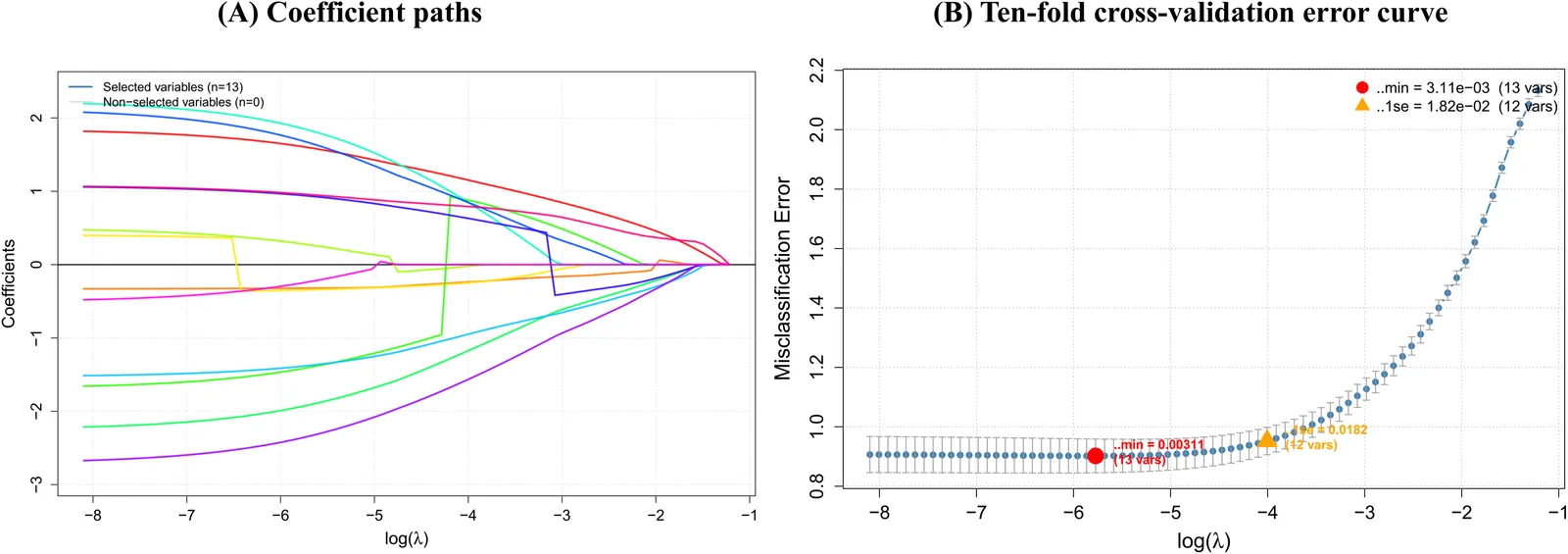
LASSO variable selection results.

During the recruitment phase, a total of 1,150 patients were initially approached. Of these, 87 were excluded for not meeting the inclusion criteria, and 50 eligible patients declined participation. Consequently, 1,013 patients consented and received the surveys. After excluding 42 incomplete or invalid responses, a final sample of 971 patients was ultimately enrolled and analyzed (valid response rate: 95.9%). The study subjects exhibited balanced age and gender distributions, with other demographic and clinical characteristics presented in Table 2. Harman’s single‑factor test on all 33 items of the MDASI and HADS showed that the first unrotated factor explained 39.7% of the total variance, which is below the common threshold of 40%, indicating that common method bias is unlikely to have artificially inflated the correlations in this study. To identify the predictors most strongly associated with latent profiles of psychosomatic symptoms in lung cancer immunotherapy patients, multinomial LASSO regression was performed on all 14 candidate variables, as shown in Fig 2 (LASSO variable selection results). The optimal penalty parameter λ.min determined by ten‑fold cross‑validation was 0.00311, and the more conservative λ.1se (within one standard error of the minimum) was 0.01822. Ultimately, 13 variables retained non‑zero coefficients under λ.min and were entered into the subsequent multinomial logistic regression model. The retained variables included: Age, Gender, Education level, Per capita monthly family income, Living Arrangements, Clinical stage, IrAEs occurred, Treatment Modalities, Tumor metastasis, ECOG, Smoking history, Drinking History, and Regular exercise habits. Only Medical Payment was excluded by LASSO. Additionally, pairwise Spearman correlations among the retained variables showed a maximum absolute value of 0.62, confirming the absence of problematic multicollinearity.

**Table 2:** Baseline characteristics of the three potential subgroups (n = 971).

Items	N (%)	C1(n = 244)	C2(n = 284)	C3(n = 443)
Age(years)				
≤59	362 (37.3%)	53	106	203
60-74	315 (32.4%)	79	94	142
≥75	294 (30.3%)	112	84	98
Gender				
Male	527 (54.3%)	121	83	323
Female	444 (45.7%)	123	201	120
Education level				
Primary school or below	443 (45.6%)	114	163	166
Junior High School	391 (40.3%)	103	85	203
High school or above	137 (14.1%)	27	36	74
Per capita monthly family income (¥)				
<5000	401 (41.3%)	140	163	98
5001-9999	360 (37.1%)	70	67	223
≥10000	210 (21.6%)	34	54	122
Living Arrangements				
Living alone	404 (41.6%)	110	155	139
With family	567 (58.4%)	134	129	304
Medical Payment				
Insurance coverage	906 (93.3%)	223	268	415
Out-of-Pocket	65 (6.7%)	21	16	28
Clinical stage				
III	409 (42.1%)	63	66	280
IV	562 (57.9%)	181	218	163
IrAEs occurred				
No	470 (48.4%)	139	209	122
Yes	501 (51.6%)	105	75	321
Treatment Modalities				
Monotherapy	396 (40.8%)	55	124	217
Combination therapy	575 (59.2%)	189	160	226
Tumor metastasis				
Yes	801 (82.5%)	213	234	354
No	170 (17.5%)	31	50	89
ECOG				
0	273 (28.1%)	33	61	179
1	317 (32.6%)	50	102	165
2	195 (20.1%)	71	62	62
3	186 (19.2%)	90	59	37
Smoking history				
No	510 (52.5%)	189	183	138
Yes	461 (47.5%)	55	101	305
Drinking History				
No	460 (47.4%)	149	113	198
Yes	511 (52.6%)	95	171	245
Regular exercise habits				
Yes	417 (42.9%)	26	87	304
No	554 (57.1%)	218	197	139

**Note:**C1: High Symptom Burden with Co-morbid Distress; C2: Emotional-Distress Dominant Phenotype; C3: Mild Adaptive Phenotype.

#### 3.2.2 Multivariate logistic regression.

To explore the factors influencing latent category assignment, this study constructed a multivariate multiclass logistic regression model using Class 3 as the reference group. To control for the false positive rate resulting from multiple comparisons, Benjamini-Hochberg FDR-corrected p-values (q-values) were calculated separately for each comparison group. Detailed results are presented in Table 3. Analysis of common predictive factors revealed that multiple shared factors significantly influence the probability of patients being assigned to Class 1 or Class 2. Regarding risk factors, smoking history, clinical stage IV, advanced age, and poor performance status all significantly increased the risk of patients being classified into Class 1 and Class 2 (all adjusted *p* < 0.05). Conversely, the absence of tumor metastasis, receiving combination therapy, living with family, experiencing irAEs, and having a habit of regular exercise were protective factors that significantly reduced the probability of patients being classified into Class 1 and Class 2 (all adjusted *p* < 0.05). In terms of specific characteristics, compared with Class 3, being female significantly increased the probability of being classified as Class 2, while having a junior high school education and a higher monthly per capita household income significantly reduced the probability of being classified as Class 2; however, these factors did not significantly predict Class 1. Furthermore, it is worth noting that when predicting Class 1, a history of alcohol consumption was significant before FDR correction but lost statistical significance after correction, showing only a marginally significant trend.

**Table 3:** Multivariate logistic regression analysis of psychosomatic symptom categories in lung cancer patients undergoing immunotherapy (n = 971).

Comparison	Variable	β	SE	OR	95% CI	P (raw)	P (FDR-adjusted)
Class 1 vs 3*							
Smoking history	No*(Ref)						
	Yes	3.337	0.860	28.126	5.215–151.8	<0.001	<0.001
Clinical stage	III*(Ref)						
	IV	4.092	0.630	59.866	17.412–205.826	<0.001	<0.001
Tumor metastasis	Yes*(Ref)						
	No	−4.097	0.674	0.017	0.004–0.062	<0.001	<0.001
Treatment Modalitie	monotherapy*(Ref)						
	combination therapy	−1.532	0.485	0.216	0.083-0.560	0.002	0.004
Age	≤59* (Ref)						
	60-74	2.789	0.342	16.265	8.320–31.798	<0.001	<0.001
	≥75	2.542	0.882	12.697	2.255–71.477	0.004	0.007
Living Arrangements	alone*(Ref)						
	With family	−1.876	0.542	0.153	0.053-0.443	0.001	0.003
IrAEs occurred	no*(Ref)						
	yes	−2.235	0.694	0.106	0.027-0.418	0.001	0.003
Regular exercise habits	no*(Ref)						
	yes	−1.876	0.585	0.153	0.049–0.482	0.001	0.003
ECOG	0*(Ref)						
	1	1.767	0.626	5.854	1.716–19.967	0.004	0.007
	2	2.124	0.761	8.366	1.884-37.132	0.005	0.008
	3	1.990	0.814	7.312	1.485–35.990	0.014	0.021
Education level	primary school or below*(Ref)						
	junior High School	−0.637	0.500	0.529	0.199–1.408	0.202	0.242
	high school or above	−0.561	0.555	0.571	0.192–1.695	0.313	0.331
Gender	male*(Ref)						
	female	0.792	0.654	2.207	0.612–7.951	0.226	0.254
Per capita monthly family income (¥)	≤5000*(Ref)						
	5001-9999	0.532	0.679	1.703	0.450–6.445	0.433	0.433
	≥10000	1.088	0.807	2.967	0.610–14.421	0.178	0.229
Drinking history	no*						
	yes	−1.053	0.513	0.349	0.128–0.954	0.040	0.055
Class 2 vs 3*							
Smoking history	No*(Ref)						
	Yes	1.226	0.301	3.41	1.890–6.140	<0.001	<0.001
Clinical stage	III*(Ref)						
	IV	1.482	0.539	4.402	1.531–12.659	0.006	0.012
Tumor metastasis	Yes*(Ref)						
	No	−2.588	0.591	0.075	0.024–0.239	<0.001	<0.001
Treatment Modalitie	monotherapy*(Ref)						
	combination therapy	−0.988	0.399	0.372	0.170-0.814	0.014	0.023
Age(years)	≤59* (Ref)						
	60-74	1.992	0.491	7.330	2.801–19.186	<0.001	<0.001
	≥75	1.254	0.568	3.505	1.151-10.672	0.027	0.037
Living Arrangements	alone*(Ref)						
	With family	−2.618	0.419	0.073	0.032-0.167	<0.001	<0.001
IrAEs occurred	no*(Ref)						
	yes	−1.223	0.563	0.294	0.097-0.888	0.030	0.039
Regular exercise habits	no*(Ref)						
	yes	−1.051	0.503	0.350	0.131–0.936	0.037	0.042
ECOG	0*(Ref)						
	1	1.368	0.531	3.927	1.387–11.122	0.010	0.018
	2	2.001	0.712	7.394	1.831–29.894	0.005	0.011
	3	2.880	0.674	17.814	4.754–66.686	<0.001	<0.001
Education level	primary school or below*(Ref)						
	junior High School	−1.281	0.416	0.278	0.123–0.628	0.002	0.006
	high school or above	−1.069	0.564	0.343	0.114–1.037	0.058	0.061
Gender	male*(Ref)						
	female	1.191	0.519	3.291	1.191–9.098	0.022	0.033
Per capita monthly family income (¥)	≤5000*(Ref)						
	5001-9999	−1.286	0.448	0.276	0.115-0.664	0.004	0.010
	≥10000	−1.124	0.535	0.325	0.114-0.927	0.035	0.042
Drinking history	no*(Ref)						
	yes	−0.232	0.450	0.793	0.328–1.916	0.606	0.606

Abbreviations: β, regression coefficient; SE, standard error; OR, odds ratio; CI, confidence interval; FDR, false discovery rate (Benjamini-Hochberg method). Reference category for the outcome variable: Class 3. Reference categories for each predictor are indicated in brackets.

The final model showed significantly better fit than a null intercept‑only model (likelihood ratio test: χ² = 245.6, df = 26, *p* < 0.001). McFadden pseudo R^2^ = 0.27, AIC = 1245.3. Overall classification accuracy was 68.5% (Class 1: 71.2%, Class 2: 62.8%, Class 3: 70.5%). Residual analysis using Pearson residuals revealed no absolute values >2, and the maximum Cook’s distance was 0.12(<1), indicating no influential outliers.

### 3.3 Correlation analysis

**Fig 3:**
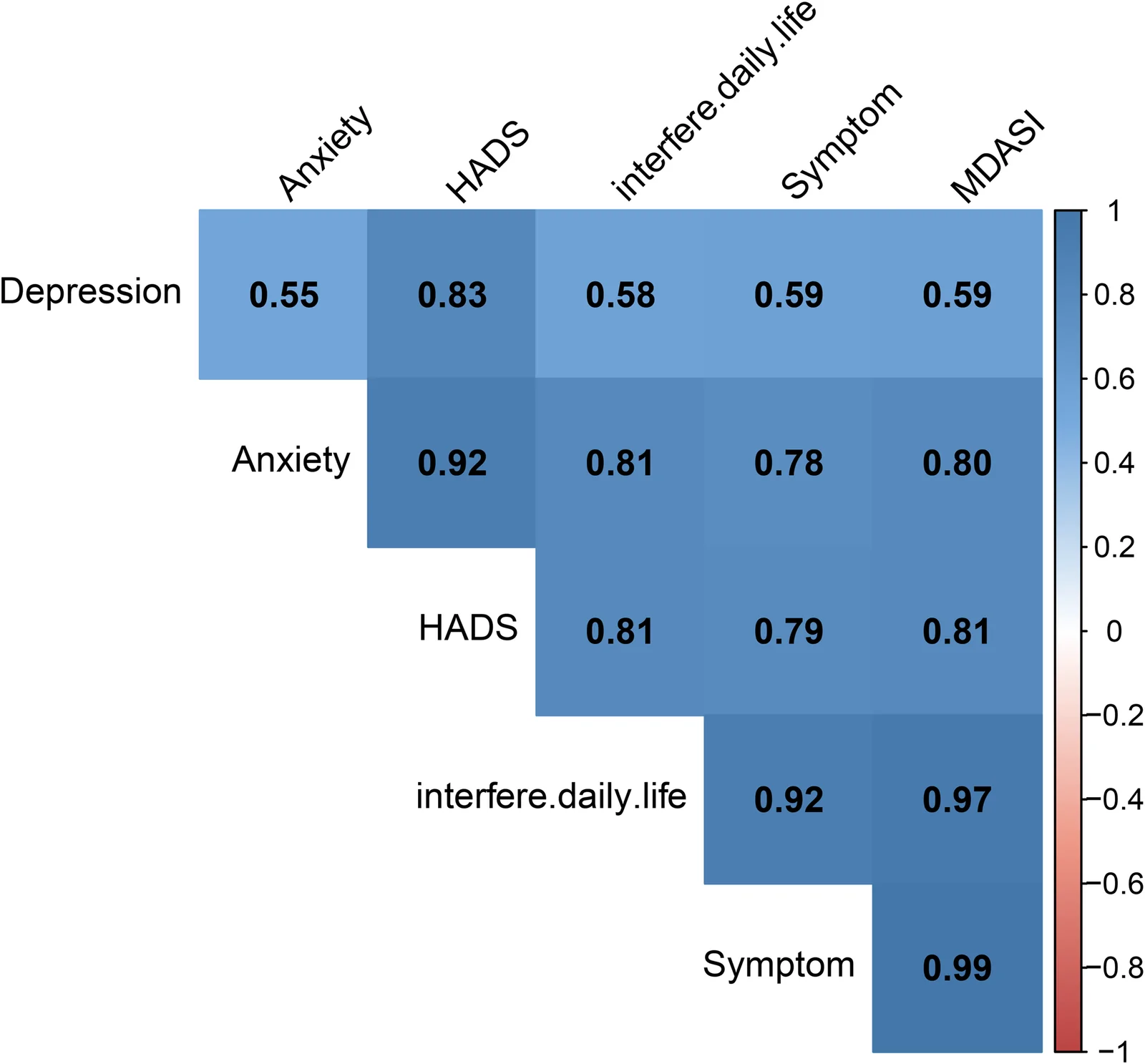
Partial correlation heatmap of symptoms, anxiety, and depression in lung cancer patients receiving immunotherapy.

After controlling for confounding factors such as age and gender, partial correlation analysis revealed (Fig 3) that Anderson core symptoms and symptom interference in lung cancer immunotherapy patients showed strong positive correlations with anxiety and mood. Among these, core symptoms exhibited the strongest correlation with symptom interference (r = 0.99), while the anxiety module correlated with both at 0.81 and 0.78, respectively. Notably, the depression module exhibited generally weaker associations with other variables, suggesting that depressive mood may be relatively independent from perceived physical symptoms and impaired daily functioning.

### 3.4 Network analysis

#### 3.4.1 Network structure.

**Fig 4:**
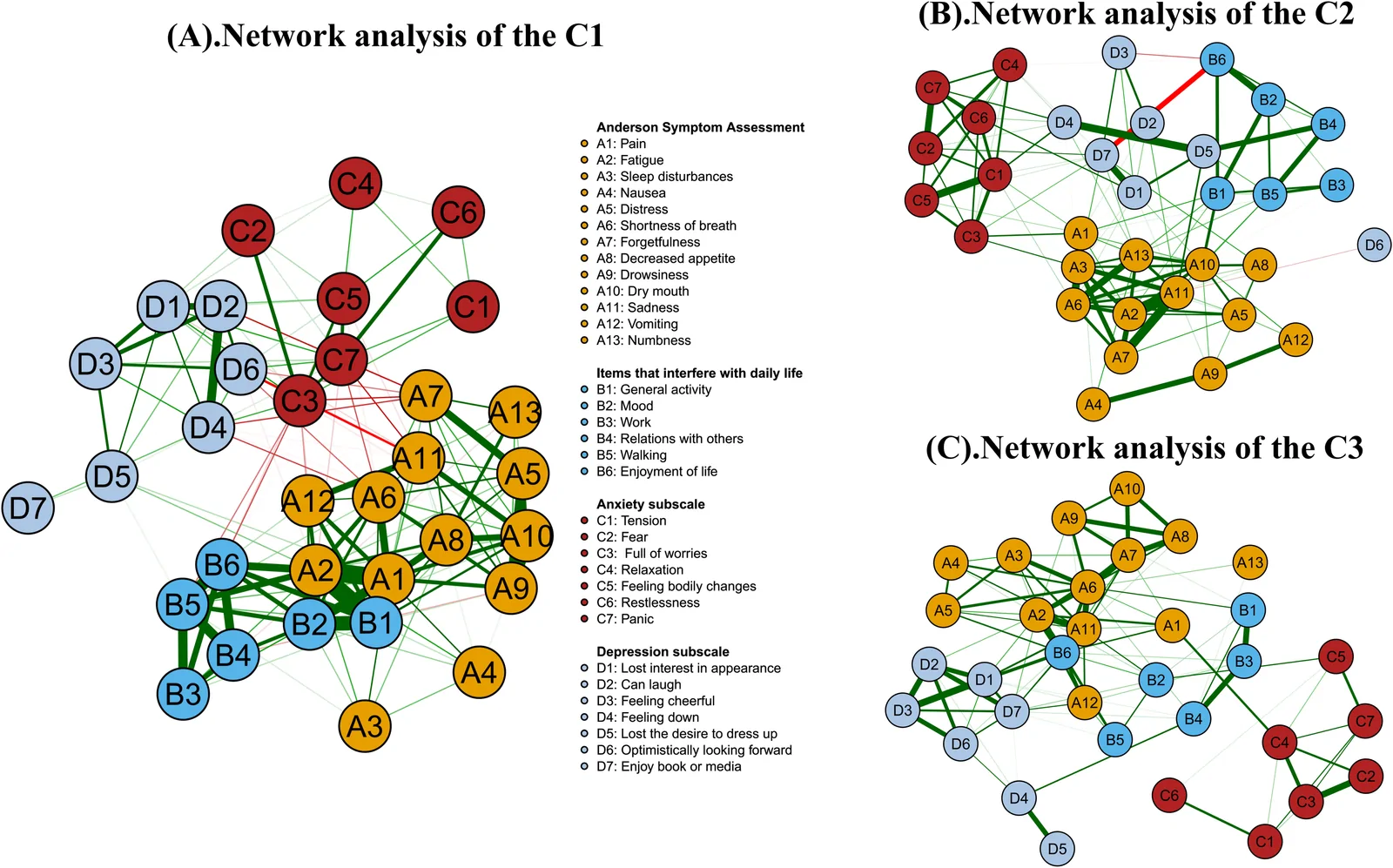
Network analysis of three profile features.

**Fig 5:**
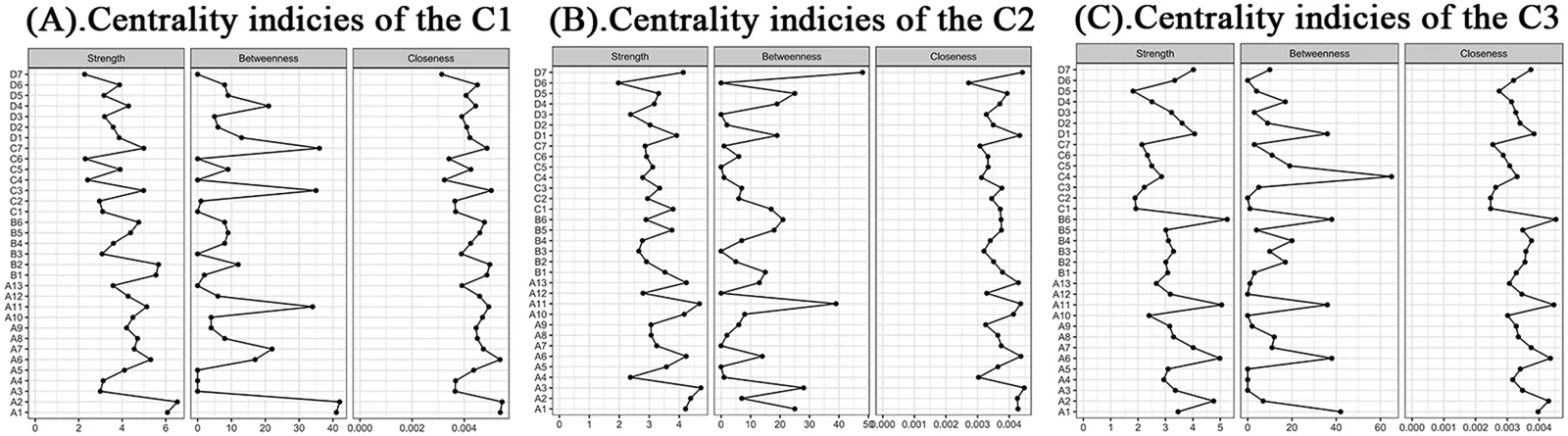
Centrality indicies of three profile features.

Based on latent profile analysis, we identified three patient subgroups (C1, C2, C3) and constructed their psychosomatic symptom networks (Fig 4). Centrality analysis (Fig 5) revealed significant differences in core symptoms and association patterns across subgroups: Subgroup C1 featured A2 (rs = 6.53) as its core symptom, with the strongest association observed between A2 and B1 (r = 0.55), suggesting fatigue may cause severe daily functional impairment; Subgroup C2 centered on A3 (rs = 4.72), with the strongest association occurring between A11 and A2 (r = 0.45), indicating a close relationship between low mood and physical fatigue in this subgroup; Subgroup C3 centered on B6 (rs = 5.25), with the strongest association between A11 and B6 (r = 0.58), highlighting the significant impact of emotional states on perceived quality of life. Overall, clusters C1 and C2 exhibited denser network connectivity, reflecting complex symptom interactions and stronger comorbid tendencies. In contrast, cluster C3 exhibited a relatively sparse network structure, indicating weaker symptom associations and a lower overall burden. For detailed information on the weight of each connection in the network, please refer to the supplementary materials: S1-S4 Tables.

**Fig 6:**
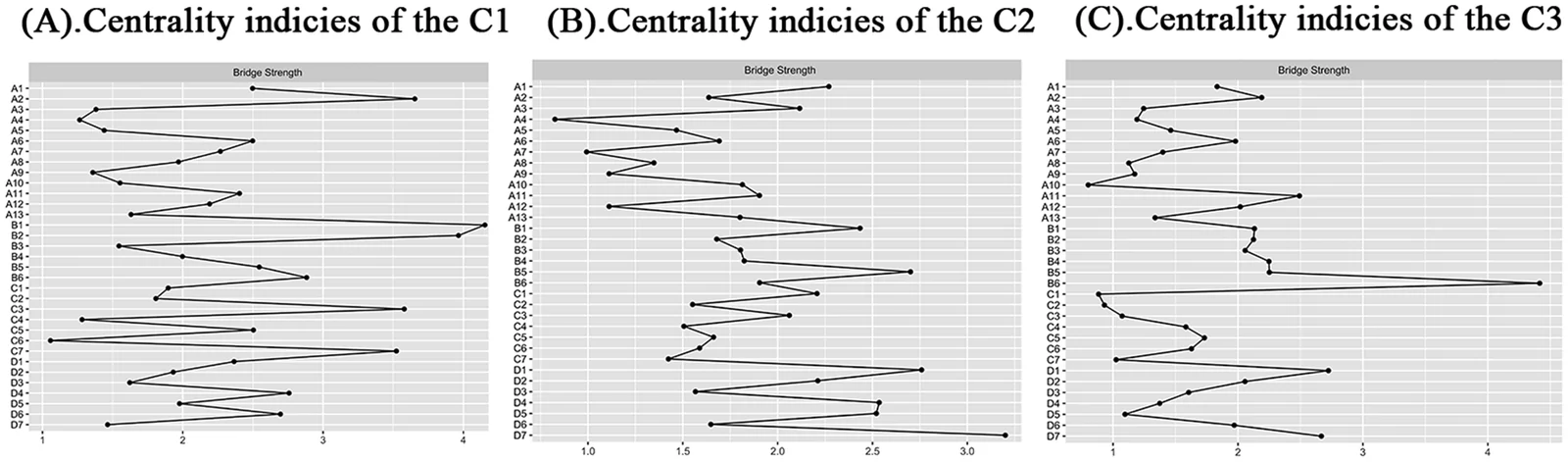
Bridge strength of three potential profile features.

The results of the bridging strength centrality analysis (Fig 6) further identified core bridging symptoms connecting different symptom dimensions. In the C1 network, B1 emerged as the most critical bridging symptom with the highest expected impact; In the C2 network, D7 was identified as the core bridging symptom; while in the C3 network, B6 played the most important bridging role. These symptoms, serving as key nodes connecting psychological and physiological dimensions, exert significant influence on maintaining the overall network structure.

#### 3.4.2 Network stability and accuracy.

**Fig 7:**
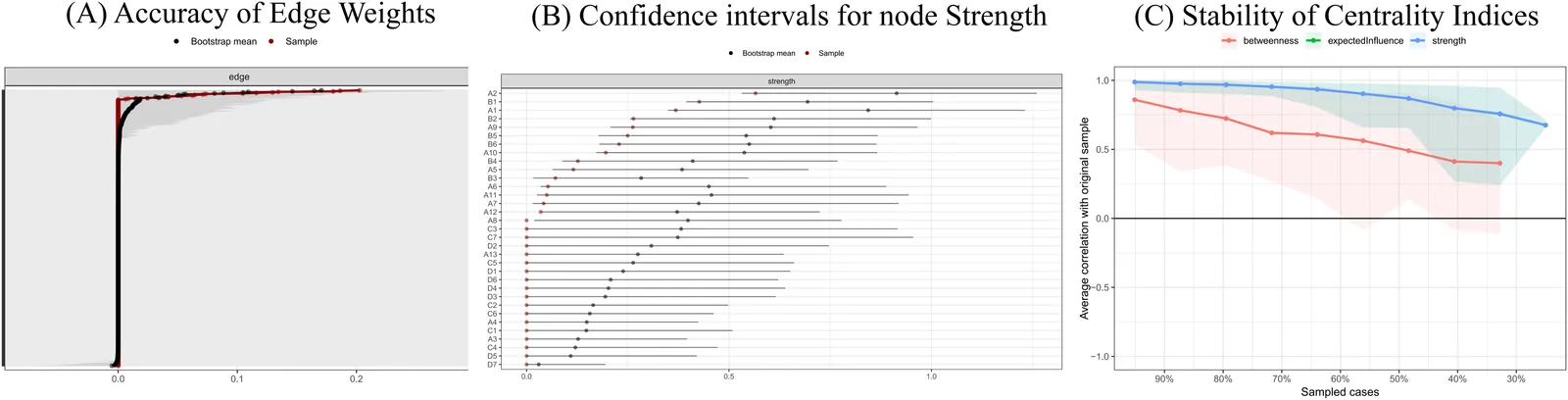
Network stability and accuracy analysis for Group C1.

**Fig 8:**
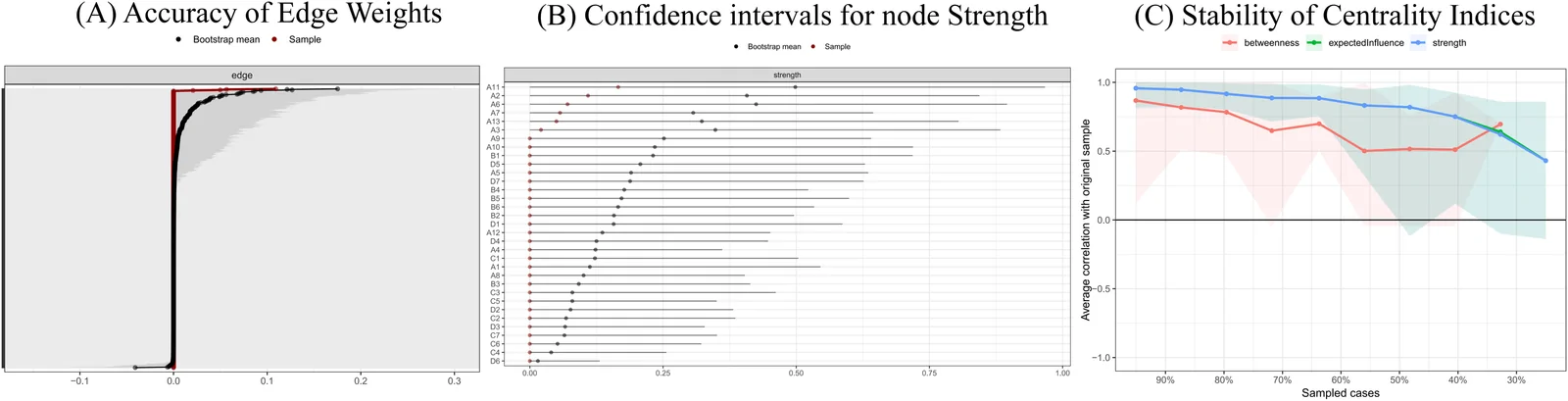
Network stability and accuracy analysis for Group C2.

**Fig 9:**
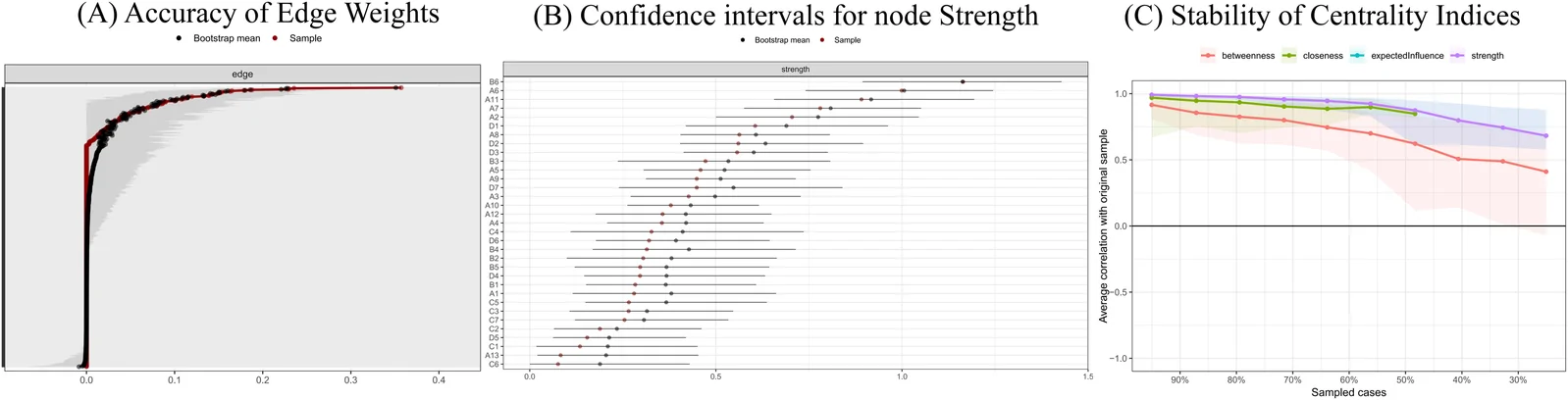
Network stability and accuracy analysis for Group C3.

As shown in Fig 7 (C1 group), the 95% bootstrapped confidence intervals for the strongest edges were narrow, indicating robust edge weight estimation. The case‑dropping bootstrap yielded CS coefficients for strength centrality of 0.672 (C1), 0.49 (C2), and 0.672 (C3), all exceeding the recommended threshold of 0.25 (with C1 and C3 > 0.5, indicating good stability). The CS coefficients for betweenness centrality were 0.361 (C1), 0.67 (C2), and 0.438 (C3), all above 0.25. Bootstrapped confidence intervals for node strength showed that high‑centrality nodes (e.g., A2 in C1) had narrower intervals, confirming the reliability of the identified core symptoms. Results for C2 and C3 are presented in Figs 8 and 9, respectively.

#### 3.4.3 Intergroup comparison of three profile networks.

**Fig 10:**
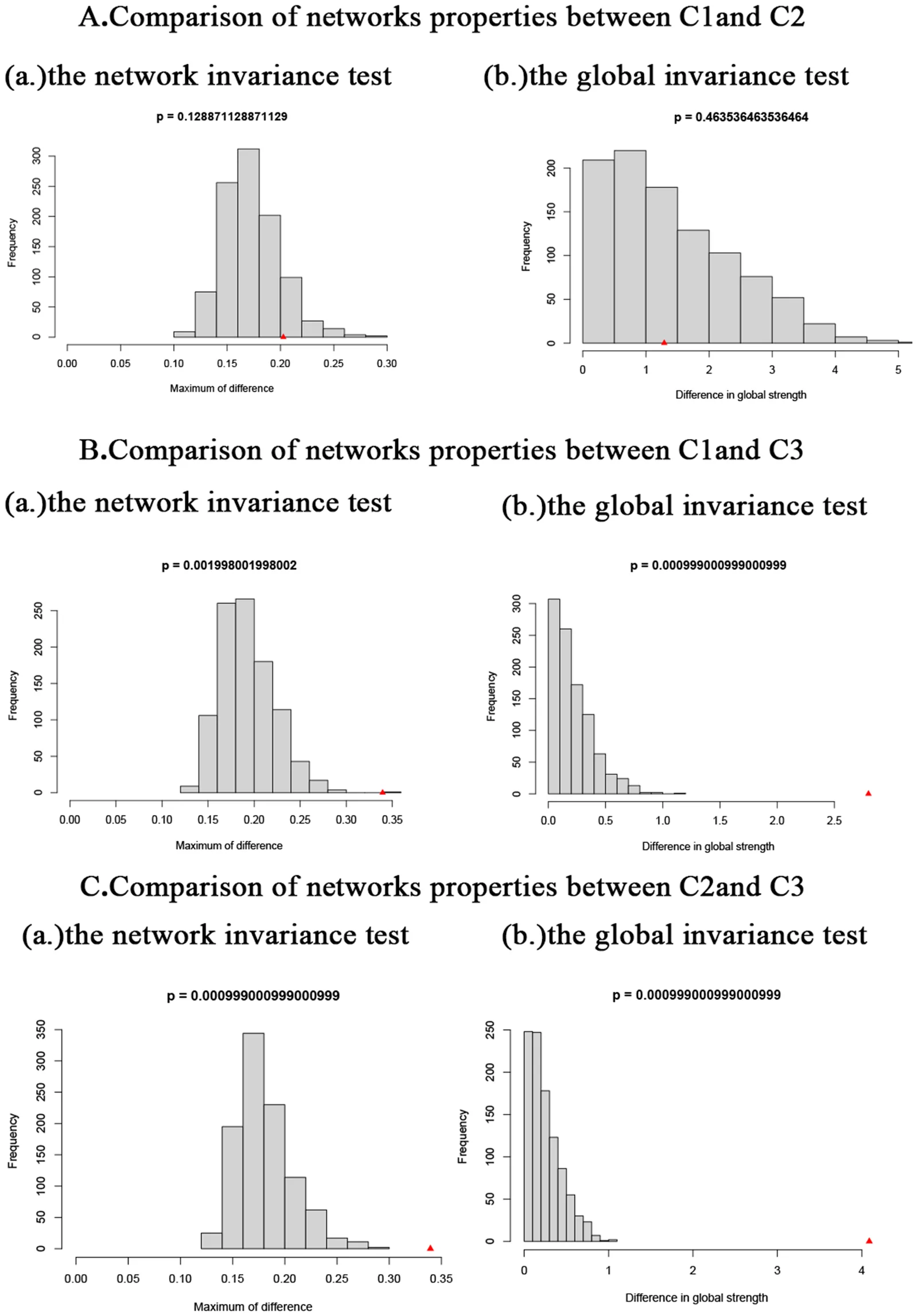
Comparison of network properties among three subgroups.

Network comparison test (NCT) results revealed no significant difference in network structure between C1 and C2 (M = 0.203, *P* = 0.104). However, statistically significant differences were observed between C1 and C3 (M = 0.339, *P* < 0.001) and between C2 and C3 (M = 0.339, *P* < 0.001). Regarding global strength, no significant difference was found between C1 and C2 (S = 1.290, *P* = 0.471). However, significant differences in global strength were observed between C1 and C3 (S = 2.30, *P* < 0.001) and between C2 and C3 (S = 4.089, *P* < 0.001). Further analysis revealed that both C1 and C2 groups exhibited higher network density than C3, with C1 demonstrating the strongest overall edge strength. Additionally, several specific connections with significant negative correlations were identified between the C1 and C2 networks (Fig 10).

### 3.5 Computer-simulated network intervention

**Fig 11:**
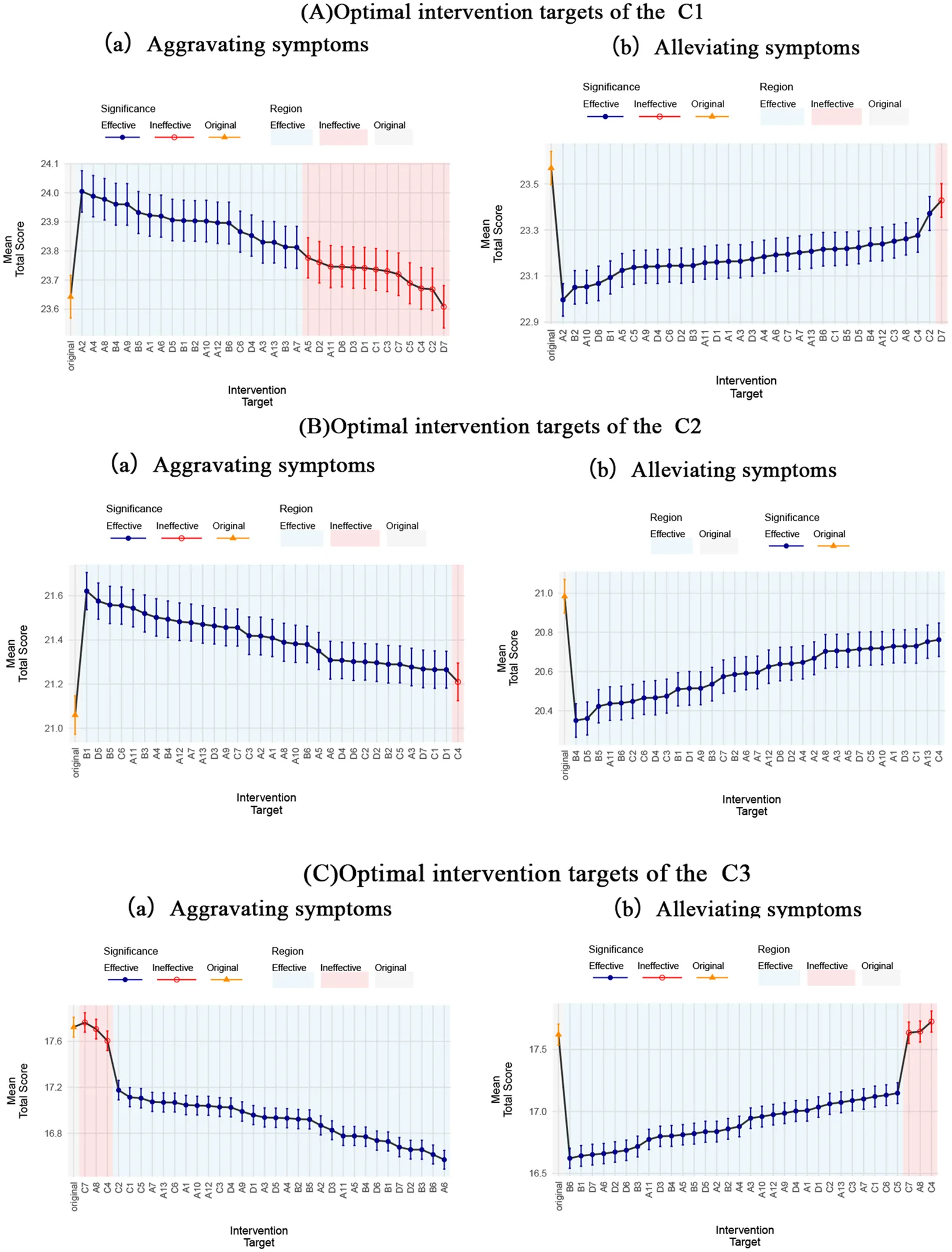
Optimal intervention targets.

To identify the most promising symptom targets for intervention, we performed computer simulations separately in the three subgroups. For each subgroup, we individually simulated the effects of improving or worsening each symptom and calculated the corresponding changes in the total symptom score. As shown in Fig 11, symptoms located in the “blue zone” (corrected *P* < 0.05) represent the most promising therapeutic targets. The simulation results identified distinct optimal targets for each subgroup: In C1, improving A2 yielded the largest reduction in total symptom score (a decrease of 2.46%). In C2, improving B4 provided the best simulated symptom relief (a decrease of 3.00%), whereas worsening B1 led to the most marked deterioration (an increase of 2.66%). In C3, enhancing B6 showed the most notable simulated improvement (a decrease of 5.68%). (Detailed data on all score changes are provided in Supplementary Material 5).

These findings suggest that intervention strategies should be tailored to each subgroup: the C1 group may benefit most from management of core physical symptoms (e.g., fatigue); the C2 group may require a dual‑pronged approach that maintains daily functioning while supporting social connections; and the C3 group may benefit from strengthening positive psychological resources. Importantly, because these computer simulations are based on cross‑sectional data, the results are hypothesis‑generating only and must be validated by future prospective studies or clinical intervention trials.

## Discussion

To our knowledge, this is the first study to employ latent profile analysis to evaluate the heterogeneity of psychosomatic symptoms in lung cancer patients receiving immunotherapy, delineating three stable and distinguishable symptom phenotypes: “High Symptom Burden with Co-morbid Distress” (C1, 25.1%), “Emotional-Distress Dominant Phenotype” (C2, 29.2%), and “Mild Adaptive Phenotype” (C3, 45.6%). This person-centered classification transcends the limitations of traditional descriptions focusing on single symptoms or symptom clusters. Mapping these the specific co-morbidity structures formed by symptoms and psychological distress across different subgroups, providing a crucial foundation for stratified and precise symptom management [42,43].

Multivariate analysis revealed that Class 1 and Class 2 were associated with distinct clinical and psychosocial factors. Patients with a smoking history, advanced clinical stage (IV), older age, poor ECOG performance status, or limited social support presented with higher risk levels for these adverse outcomes. Conversely, combination therapy, regular exercise, living with family, and the occurrence of mild immune‑related adverse events (irAEs) were associated with lower risk levels. These findings suggest that multidimensional, phenotype‑specific interventions may have clinical utility. Symptom burden appeared to be closely intertwined with physiological, treatment‑related, psychosocial, and prognostic factors. Physiological correlates: Smoking history was the strongest risk‑associated factor for both phenotypes, and its relationship with symptom burden may involve pre‑activation of the immune system and increased susceptibility to irAEs, which in turn were accompanied by higher symptom burden during immunotherapy [44,45]. Older age (≥75 years) was particularly associated with the high‑burden phenotype, possibly reflecting the cumulative impact of multimorbidity and diminished physiological reserve. Treatment‑related correlates: Poor ECOG performance status (≥2) was strongly associated with both phenotypes, with ECOG 3 showing a particularly notable association with emotional distress. Combination therapy was associated with lower risk, which may suggest better disease control or integrated supportive care. Notably, mild irAEs showed an association with lower risk in the high‑burden phenotype. This finding is consistent with previous evidence suggesting that mild irAEs may serve as a clinical surrogate for effective immune activation, without being accompanied by severe toxicity [46]. Psychosocial and prognostic context: Living with family and regular exercise were identified as important protective correlates, possibly by enhancing coping capacity and mitigating cancer‑related fatigue. Lower education level (elementary school or below) was associated with increased risk of emotional distress, while junior high school education and middle‑income status were associated with lower risk, consistent with the widely held view in the literature that lower socioeconomic status is associated with poorer cancer outcomes [47]. Advanced stage (IV) remained the factor most strongly associated with high symptom burden, and female gender was a significant correlate of emotional distress, consistent with established sex‑based differences in psychological morbidity [26]. In summary, clinical practice may consider moving toward a multidimensional, individualized management approach. For high risk patients particularly those with a smoking history, poor ECOG status, and lack of family support multidisciplinary teams could consider integrating proactive symptom monitoring, psychological support, and tailored exercise prescriptions. Future prospective studies are warranted to further validate the clinical significance of mild irAEs and to explore phenotype‑based management pathways in lung cancer immunotherapy.

To our knowledge, this is the first combined LPA and network analysis study targeting lung cancer immunotherapy patients, exploring LPA-based networks through comparative network testing and computer simulation interventions. Different phenotypic networks exhibit distinct core and bridging symptoms.

In Group C1, symptom network analysis revealed that A2 occupied the network core, forming dense connections with somatic, functional, and psychological symptoms; while B1 served as a key bridging symptom. This structure suggests that the “fatigue-activity limitation” pathway is the core link sustaining this high-burden symptom network, potentially driving a positive feedback loop of “declining physiological function-increased psychological distress-worsening physical symptoms” [48]. Notably, fatigue is not only a common symptom in immunotherapy (prevalence 12%−37%) [49,50], but research also suggests it may serve as a potential biomarker for disease progression or poor treatment tolerance [51]. Thus, from a network centrality perspective, fatigue is not merely a symptom burden requiring management, but a critical lever point for intervening in the network of multiple symptoms. Based on network theory [52], synergistic intervention targeting the core node and bridging node represents the optimal therapeutic approach. Integrating evidence-based exercise therapy with behavioral activation strategies [53,54] within the NCCN guidelines framework is recommended. This approach holds promise for simultaneously improving physical function and psychological distress, thereby providing a precise intervention pathway to break the high-burden cycle observed in C1 group patients.

The symptom network of the C2 group exhibited a distinct topological structure centered on A3 and D7. The strong association between A11 and A2 indicates a high covariation between emotional and physical symptoms in these patients. From a psychoneuroimmunology perspective, it has been hypothesized that sleep disturbance may dysregulate the HPA axis and modulate pro-inflammatory cytokines (e.g., IL-6, TNF-α), which have been linked to anxiety, depression, and fatigue in previous studies [55–57]. This possible pathway has been proposed as a “neuroinflammation

behavior” loop in the literature. Notably, our network analysis highlights the structural role of anhedonia [58,59] it is not only a core feature of depression, but also appears to serve as a key bridge, potentially connecting localized emotional distress to broader quality-of-life impairments. This suggests, albeit speculatively, that anhedonia might be involved in the co-occurrence of emotional distress and functional decline. Therefore, interventions for the C2 group may extend beyond single-symptom management toward a dual-pathway approach: regulating core drivers (e.g., improving sleep through CBT-I) alongside blocking bridging pathways (e.g., rebuilding pleasurable experiences through mindfulness-based behavioral activation). Future research could explore phenotype-specific psychosocial support models targeting anhedonia, and incorporate biomarkers (e.g., inflammatory markers) to evaluate intervention responses.

In Group C3, although the overall symptom burden was the lightest, network analysis revealed a highly concentrated core pattern: B6 served a dual role as both a core symptom and a bridge symptom. The strongest symptom association in this group occurred between A11 and B6, suggesting that even in patients with well-controlled physical symptoms, emotional state may be a key factor influencing their perception of quality of life. This network structure has important clinical implications. The symptom network in Group C3 is no longer driven by multiple somatic symptoms but rather revolves around “emotional well-being.” Therefore, for patients in Group C3, the focus of clinical attention can shift from symptom management to enhancing positive psychological resources. Interventions may emphasize behavioral activation and the reconstruction of positive experiences. Given that this study employed a cross-sectional design, the above interpretations remain speculative and require validation through future prospective studies.

Network comparison revealed that C1 and C2 exhibited similar structures, while both differed significantly from C3 (*P* < 0.001). Further analysis showed that networks in the C1 and C2 groups were more densely connected. While early network theories suggest that high connectivity may reflect a self-reinforcing “pathological steady state” among symptoms [60], this hypothesis remains heavily debated. In contrast, the network structure of the C3 group was sparse, with weak inter-symptom connections, which may be characteristic of a milder symptom profile. Although previous studies have proposed that network density might hold discriminative value [61], recent methodological literature cautions against overinterpreting global connectivity. Differences in network density can be highly sensitive to the scaling (variance) of the variables, sample size variations, and the specific estimation methods utilized (e.g., regularization techniques) [28,62,63]. Despite these methodological nuances, the observed structural differences suggest that clinical assessment could benefit from considering connectivity patterns alongside overall severity. Based on these structural differences and our simulation-derived hypotheses, intervention strategies could be considered for tailored management: for the C1 and C2 groups, targeting core and bridge symptoms may help reduce overall system activity; for the C3 group, maintaining its sparse network structure could be a goal to prevent deterioration. This highlights a potential shift in symptom management from traditional “symptom alleviation” toward structure-informed precision approaches, pending empirical validation.

Finally, cross‑sectional‑based computer‑simulated intervention analyses identified A2 in Group C1 and B6 in Group C3 as their respective potential optimal intervention targets under the simulated conditions, which is consistent with the core and bridge symptoms identified by prior network analysis. This finding supports the hypothesis that intervening on the most influential nodes in a network might most effectively perturb and alleviate the overall symptom burden. Research in breast cancer populations also confirms [64] that optimal targets identified through computer simulation highly overlap with core symptoms identified by traditional network models, reinforcing the reliability and forward‑looking nature of combining network analysis with computational simulation for identifying high‑value intervention targets. However, the simulation results for Group C2 revealed a more complex phenomenon worthy of further exploration: although network analysis confirmed the central role of “sleep disorders (A3),” the simulated intervention showed that improvements in “interpersonal relationships (B4)” led to better overall symptom relief. This seemingly contradictory result actually reflects two conceptually distinct metrics in network analysis. Core symptoms (identified by centrality metrics) describe the node most strongly associated with a given symptom within the network, reflecting its prominent position in the existing network structure; whereas optimal intervention targets (identified by simulated perturbations) assess the systemic impact that altering a specific symptom can produce, that is, “whether changing one symptom can drive overall improvement” [65]. Previous studies have also confirmed that core symptoms and optimal intervention targets do not always align [66,67]. With respect to Group C2, one speculative interpretation is as follows: although sleep disturbance exhibits high centrality, it might be a “receiver” symptom, easily influenced by factors such as emotional distress and social isolation, yet difficult to improve through isolated intervention. In contrast, B4 may occupy a position that links psychological distress with social functioning and quality of life. Improving interpersonal relationships could influence multiple domains, potentially reducing loneliness, enhancing social support, and alleviating emotional distress, which might in turn indirectly associate with improvements in sleep and other downstream symptoms. This interpretation is consistent with the biopsychosocial model, though it remains speculative given the cross‑sectional nature of our data.

In summary, the results of the computer simulation based intervention in this study provide a hypothesis-generating direction for symptom management in lung cancer patients undergoing immunotherapy: in future prospective clinical intervention trials, targets with high perturbation effects validated by simulation could be prioritized based on patients’ symptom phenotypes to achieve personalized treatment that goes beyond generic regimens. However, it is important to emphasize that all of the above conclusions are based on cross-sectional data and should not be interpreted as causal predictions; they require further validation through longitudinal or experimental studies.

### Limitations

Although this study provides new insights into symptom management for lung cancer patients undergoing immunotherapy through latent profile analysis and symptom network analysis, several limitations remain. First, all symptom data were collected via self-reported questionnaires, which may be subject to recall bias, social desirability bias, and individual perceptual differences, and lack objective clinical assessment. Second, the cross-sectional design can only reflect the structure of the symptom network at a specific point in time and cannot track the dynamic evolution of symptoms and core/bridge symptoms as treatment progresses and the disease advances; furthermore, estimates of network parameters may be unstable due to limited sample sizes in subgroups and methodological choices. Third, computer-simulated interventions based on cross-sectional data represent only hypothetical perturbations of static correlation structures, rather than causal predictions. Fourth, single-center convenience sampling may limit the generalizability of the results. Fifth, this study did not collect baseline mood data or a systematic psychiatric history prior to immunotherapy, nor did it include detailed clinical information (such as ICI type, treatment cycles, and irAE characteristics) or immune-related biomarkers, thereby limiting an in-depth analysis of the mechanisms underlying symptom heterogeneity. Sixth, the confidence intervals for some effect estimates in the multivariate regression analysis are relatively wide, possibly due to small subgroup sample sizes or moderate multicollinearity among variables; these results should be interpreted with caution and validated in larger samples. In summary, all findings are hypothesis-generating and require further validation through prospective longitudinal studies and intervention trials.

## Conclusion

By combining latent profile analysis with symptom network analysis, this study identified three distinct psychosomatic symptom phenotypes in lung cancer immunotherapy patients: high symptom burden with comorbid distress, emotional distress‑dominant, and mildly adaptive. Network analysis further revealed subtype‑specific differences in core symptoms, bridge symptoms, and network architecture. Based on cross‑sectional data, computer‑simulated interventions provided hypothesis‑generating evidence for precision targets unique to each phenotype. These findings align with network theory, suggesting that targeting central nodes may yield the greatest systemic benefits. Moving beyond traditional single‑symptom or cluster approaches, this study offers an exploratory, mechanism‑driven roadmap: fatigue management for the high‑burden subtype, combined sleep and social function strategies for the emotion‑dominant subtype, and positive psychological resource cultivation for the mildly adaptive subtype. All simulation‑based findings require validation in longitudinal or experimental studies. Future research should adopt prospective designs to translate these computational insights into improved clinical outcomes.

## Supporting information

S1 Fig Distribution plots for 3–5 potential features. (TIF)

S1 Table Detailed connection weights for each connection in the C1 group network. (CSV)

S2 Table Detailed connection weights for each connection in the C2 group network. (CSV)

S3 Table Detailed connection weights for each connection in the C3 group network. (CSV)

S4 Table Centrality strength for all symptoms. (XLSX)

S5 File Complete simulated data for the three group networks. (DOCX)
