# Supplementary material for: A Latent profile and network intervention analysis of psychosomatic symptoms in lung cancer immunotherapy patients
Source: PLoS One. 2026 Sep 9;21(9):e0357460. doi: 10.1371/journal.pone.0357460 (PMC13557409; doi:10.1371/journal.pone.0357460)
Supplement: Supplementary file 1 [file pone.0357460.s006.docx]

High Symptom Burden with Co-morbid Distress

Aggraviating	Alleviating

Emotional-Distress Dominant Phenotype

Aggraviating	Alleviating

Mild Adaptive Phenotype

Aggraviating	Alleviating
